# Commencement

**Published:** 1877-04

**Authors:** 


### COMMENCEMENT.

The second annual commencement of the Dental College of the University of Michigan was held on the 28th of March, when the Degree of Doctor of Dental Surgery was conferred upon the following named gentlemen, viz:

M. Holland, M. D., St. Joseph, Mich.
Wm. G. Stowell, Manchester, Mich.
P. J. Kester, Chicago, Ill.
P. McGreger, Ann Arbor, Mich.
H. V. Jackson, Ann Arbor, Mich.
G. W. Irvin, Lima, Ohio.
Geo. S. Shattuck, Bell Plain, Iowa.
S. B. Hartman, Ft. Wayne, Ind.

The address to the class was delivered by Dr. A. T. Metcalf, of Kalamazoo, Mich.

The occasion was a very pleasant and interesting one; one not soon to be forgotten, especially by those who took part.

